# Studies on the Inclusion Complexes of Daidzein with β-Cyclodextrin and Derivatives

**DOI:** 10.3390/molecules22122183

**Published:** 2017-12-08

**Authors:** Shujing Li, Li Yuan, Yong Chen, Wei Zhou, Xinrui Wang

**Affiliations:** 1 Beijing Advanced Innovation Center for Food Nutrition and Human Health, Beijing Technology and Business University, Beijing 100048, China; 10011316015@st.btbu.edu.cn (L.Y.); zhouw@th.btbu.edu.cn (W.Z.); wangxinrui@th.btbu.edu.cn (X.W.); 2 Department of Chemistry, School of Science, Beijing Technology and Business University, Beijing 100048, China; 3 Key Laboratory of Photochemical Conversion and Optoelectronic Materials, Technical Institute of Physics and Chemistry, Chinese Academy of Sciences, Beijing 100190, China; chenyong@mail.ipc.ac.cn

**Keywords:** daidzein, cyclodextrin, inclusion complex, antioxidant activity

## Abstract

The inclusion complexes between daidzein and three cyclodextrins (CDs), namely β-cyclodextrin (β-CD), methyl-β-cyclodextrin (Me-β-CD, DS = 12.5) and (2-hydroxy)propyl-β-cyclodextrin (HP-β-CD, DS = 4.2) were prepared. The effects of the inclusion behavior of daidzein with three kinds of cyclodextrins were investigated in both solution and solid state by methods of phase-solubility, XRD, DSC, SEM, ^1^H-NMR and 2D ROESY methods. Furthermore, the antioxidant activities of daidzein and daidzein-CDs inclusion complexes were determined by the 1,1-diphenyl-2-picryl-hydrazyl (DPPH) method. The results showed that daidzein formed a 1:1 stoichiometric inclusion complex with β-CD, Me-β-CD and HP-β-CD. The results also showed that the solubility of daidzein was improved after encapsulating by CDs. ^1^H-NMR and 2D ROESY analyses show that the B ring of daidzein was the part of the molecule that was most likely inserted into the cavity of CDs, thus forming an inclusion complex. Antioxidant activity studies showed that the antioxidant performance of the inclusion complexes was enhanced in comparison to the native daidzein. It could be a potentially promising way to develop a new formulation of daidzein for herbal medicine or healthcare products.

## 1. Introduction

**Figure 1:**
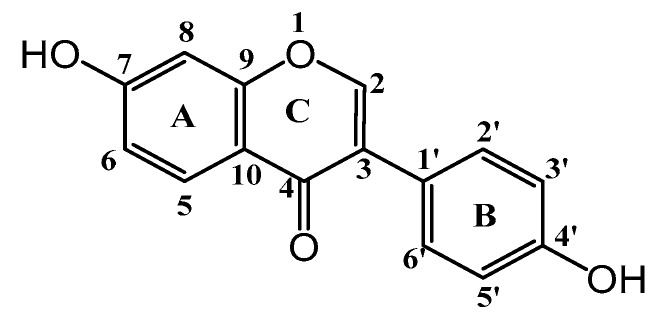
Chemical structure of daidzein.

Daidzein (Figure 1) is one of the major isoflavone compounds and exists widely in soybeans [1]. The present study shows that daidzein possesses multiple biological and pharmacological properties such as antioxidant [2,3], anticancer [4,5], anti-inflammatory [6,7], neuroprotective [8], protective treatment of cardiovascular diseases [9], and autoimmune diseases [10]. However, its use in medicines and in functional food ingredients is limited because of its poor solubility and low bioavailability. Various techniques, such as incorporation into a hydrophilic vehicle [11], phosphorylated daidzein [12], and glycosylation [13], etc., have been developed to improve its water solubility and stability. The formation of an inclusion complex with cyclodextrins (CDs) is another promising way to solve this problem.

Cyclodextrins (CDs) are cyclic oligosaccharides built up from glucopyranose units linked by α-1,4 bonds, thus forming a truncated cone. There are three principal types of natural CDs, also known as first-generation CDs: α-CD, β-CD, and γ-CD. These CDs consist of six, seven, and eight glucopyranose units, respectively. β-CD is the most commonly used in pharmaceutical formulations due to its non-toxicity, biodegradability, and its reasonable cost [14]. However, the application of unmodified β-CD is limited, owing to its poor water solubility. Accordingly, modified β-CDs have been synthesized and used, such as methylated-β-cyclodextrin (Me-β-CD) and (2-hydroxy)propyl-β-cyclodextrin (HP-β-CD) [15,16]. The special cone-shaped structure of CDs enables them to enclose the hydrophobic molecules that form the inclusion complexes. As a result of the preparation of the inclusion compound, multiple modifications are gained in the properties of guest molecules, such as improvement of the dissolution of insoluble substances [17,18], stabilization of photosensitive substances [19], and a controlled release of drugs [20,21]. The CDs and their inclusion complexes are used as additives in the drug, food, packaging, cosmetics, and textile industries [22,23]. 

Some studies have evaluated the improvement of daidzein and genistein solubility by a complexation with HP-β-CD at different host–guest molar ratios [24]. Later, Yatsu [25] reported on the multiple complexations of CDs with soy isoflavones, present in an enriched fraction. Although these studies have demonstrated the feasibility of obtaining inclusion complexes with daidzein, none of them evaluated the inclusion behavior of daidzein with different CDs. Therefore, in this present work, we evaluate the inclusion behavior of daidzein with β-CD, Me-β-CD, and HP-β-CD. The stoichiometric ratios and stability constants describing the extent of formation of the complexes were determined by phase-solubility measurements and Job’s method. The inclusion complexes were prepared by the freeze-drying method and were further characterized by X-ray diffraction (XRD), thermogravimetric (TG), differential scanning calorimetry (DSC), scanning electron microscopy (SEM), 1H-nuclear magnetic resonance spectroscopy (^1^H-NMR ) and two-dimensional rotational frame nuclear overhauser effect spectroscopy (2D ROESY). Meanwhile, the antioxidant activities of daidzein and the inclusion complexes were also investigated by the 1,1-diphenyl-2-picrylhydrazyl (DPPH) radical scavenging activity assay. 

## 2. Results and Discussion 

### 2.1. Phase-Solubility Study

**Figure 2:**
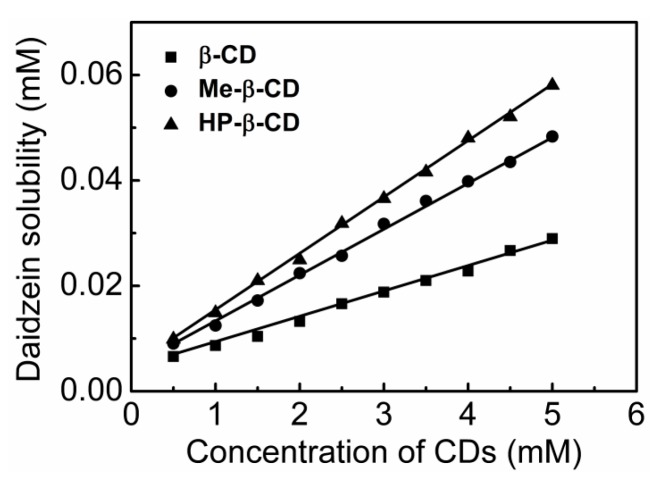
Phase-solubility diagrams of daidzein with β-cyclodextrin (β-CD), methyl-β-cyclodextrin (Me-β-CD), or (2-hydroxy)propyl-β-cyclodextrin (HP-β-CD) at 25 °C.

Phase-solubility analysis of daidzein with β-CD, Me-β-CD, and HP-β-CD was studied by the method of Higuchi and Connors [26] in an aqueous solution at 25 °C. The phase-solubility diagram is a widely useful method for the evaluation of the inclusion interaction of CDs complexation with poorly water-soluble molecules, as well as the determination of the stability constants (Ks) in the complexes formation. As shown in Figure 2, the aqueous solubility of daidzein increased linearly with the increasing CDs concentration within the studied concentration range. Based on Higuchi and Connors′s theory, these three linear host–guest correlations could be classified as A_L_ type, indicating that a 1:1 stoichiometry of the complexes exists between daidzein and the three different CDs studied. The calculated apparent stability constant (Ks, M^−1^) of daidzein-β-CD, daidzein-Me-β-CD and daidzein-HP-β-CD, was 776 M^−1^, 1418 M^−1^, and 1802 M^−1^, respectively. The higher apparent stability constants of daidzein-Me-β-CD and daidzein-HP-β-CD can be attributed to the opening enlargement of native β-CD and the destruction of the strong intramolecular hydrogen bond network by the methyl and hydroxypropyl substitutions. This destruction causes guest molecules to easily access the CD’s cavity and give a higher stability constant. The finding that Me-β-CD or HP-β-CD increases the binding capacity for flavonoids has been previously reported [27,28]. Additionally, the solubility of daidzein was significantly increased (4.8-fold, 8.1-fold, and 9.7-fold at 5 mM of β-CD, Me-β-CD, and HP-β-CD) compared to the absence of CDs, which indicated the solubilizing potential for daidzein by CDs. 

The stoichiometry of the complex formation between daidzein and CDs was also determined by Job’s method (see Supplementary data Figures S1–S3). As shown in the figures, the maximum peak was observed at R = 0.5, which indicates the formation of 1:1 inclusion complexes between daidzein and β-CD, Me-β-CD or HP-β-CD, in accordance with the phase solubility study. 

### 2.2. XRD Studies

**Figure 3:**
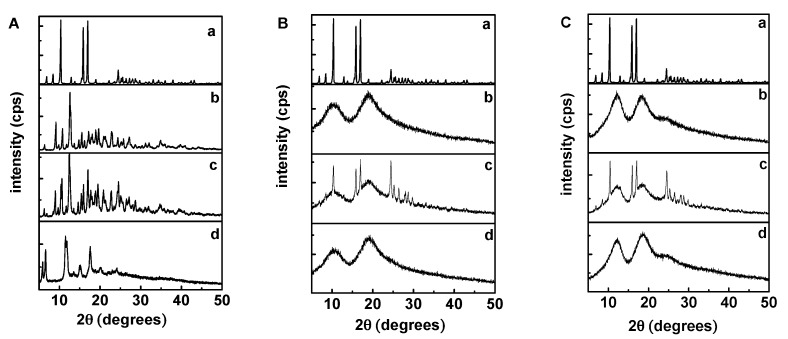
XRD patterns: (**A**) (a) daidzein, (b) β-CD, (c) daidzein/β-CD physical mixture, (d) daidzein-β-CD inclusion complex; (**B**) (a) daidzein, (b) Me-β-CD, (c) daidzein/Me-β-CD physical mixture, (d) daidzein-Me-β-CD inclusion complex; (**C**) (a) daidzein, (b) HP-β-CD, (c) daidzein/HP-β-CD physical mixture, (d) daidzein-HP-β-CD inclusion complex.

The powder X-ray diffraction patterns (XRD) is an effective method for the analysis of CDs and their inclusion complexes in the powder or microcrystalline state [29,30]. The formation of an inclusion complex between CDs and a crystalline guest means that the latter would no longer exist in the crystalline state and consequently, the diffraction pattern of the complex would not be a simple superposition of those of the two components. As indicated in Figure 3, the XRD patterns of daidzein and β-CD displayed numerous sharp peaks, characteristic of its crystallinity, whereas that of Me-β-CD and HP-β-CD showed two broad peaks, consistent with its amorphous nature. The XRD of the physical mixture of daidzein and CDs was a superposition of the patterns of the components, confirming that no chemical association had occurred between daidzein and CDs. In addition, both kept their original physical characteristics. In contrast, the XRD spectra of daidzein-β-CD, daidzein-Me-β-CD, and daidzein-HP-β-CD inclusion complexes are amorphous and show halo patterns, indicating the formation of an inclusion complex between β-CD (or Me-β-CD, HP-β-CD) and daidzein.

### 2.3. Thermal Analysis 

The thermal properties of daidzein, CDs, and daidzein-CDs inclusion complexes were studied by thermogravimetric (TG) methods (see Supplementary data Figures S4–S6). A systematic analysis of the TG curves showed that daidzein decomposed at ca. 315 °C, β-CD at ca. 298 °C, Me-β-CD at ca. 290 °C, and HP-β-CD at ca. 300 °C. In contrast, the decomposition temperature of the daidzein-β-CD, daidzein-Me-β-CD, and the daidzein-HP-β-CD inclusion complex was ca. 296 °C, 292 °C and 299 °C. These results indicate that the daidzein-CDs inclusion complexes were formed [31].

**Figure 4:**
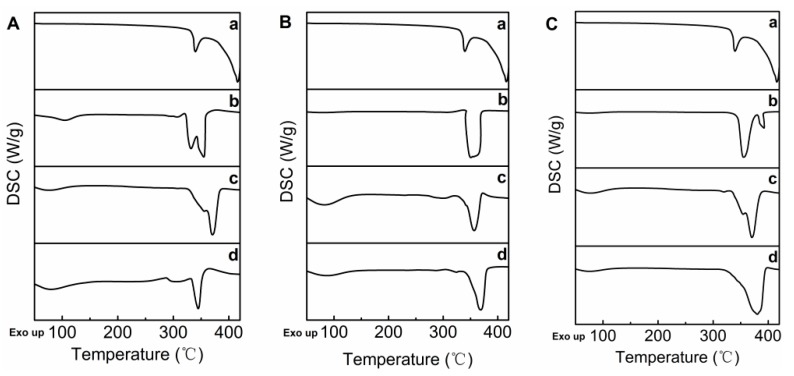
DSC thermograms: (**A**) (a) daidzein, (b) β-CD, (c) daidzein/β-CD physical mixture, (d) daidzein-β-CD inclusion complex; (**B**) (a) daidzein, (b) Me-β-CD, (c) daidzein/Me-β-CD physical mixture, (d) daidzein-Me-β-CD inclusion complex; (**C**) (a) daidzein, (b) HP-β-CD, (c) daidzein/HP-β-CD physical mixture, (d) daidzein-HP-β-CD inclusion complex.

The differential scanning calorimetry (DSC) thermogram provided further information about the thermal properties of daidzein-β-CD, daidzein-Me-β-CD, and the daidzein-HP-β-CD inclusion complex [28]. As shown in Figure 4, daidzein displayed one sharp endothermic peak at 339 °C. In contrast, the DSC curves of β-CD, Me-β-CD, and HP-β-CD had an endothermic peak at 331 °C, 349 °C and 355 °C, respectively. The DSC thermogram of the physical mixture is basically a combination of two components, with the daidzein peaks being only faintly observable due to the lower proportions that it had in the physical mixture. However, in the DSC curves of daidzein-β-CD, daidzein-Me-β-CD, and daidzein-HP-β-CD inclusion complexes, the endothermic peaks were shifted to 345 °C, 368 °C and 380 °C, suggesting that an inclusion structure was formed between the host–guest molecules. These results further confirmed the formation of an inclusion complex between daidzein and CDs. 

### 2.4. SEM Studies

**Figure 5:**
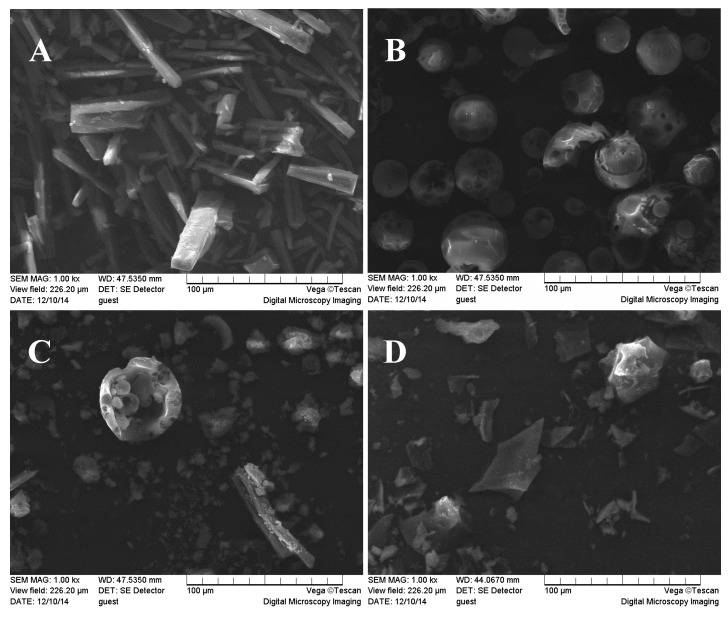
Scanning electron microphotographs: (**A**) daidzein; (**B**) HP-β-CD; (**C**) daidzein/HP-β-CD physical mixture; (**D**) daidzein-HP-β-CD inclusion complex.

Scanning electron microscopy was also a useful method to study the structure of the materials [32,33]. Figure 5 shows the SEM photographs of daidzein, HP-β-CD, their physical mixture, and their inclusion complex. Pure daidzein existed in columnar crystal with medium dimensions and HP-β-CD appeared as a spherical shape with cavity structures. The physical mixture of daidzein with CDs revealed that the characteristic crystals of daidzein and the spheres of HP-β-CD both existed separately, indicating that the two components existed in their original individual forms. In contrast, the daidzein-CDs inclusion complexes appeared as a plate-like crystal structure and were quite different from the sizes and shapes of daidzein and CDs. This observation confirmed the formation of the inclusion complex between daidzein and HP-β-CD. In a similar test, the daidzein-β-CD and daidzein-Me-β-CD appeared to be quite different from the sizes and shapes of β-CD, Me-β-CD and daidzein, respectively, which is a strong indication of an inclusion complex formation (see in the Supplementary data Figures S7 and S8).

### 2.5. ^1^H-NMR and 2D NMR

Further evidence supporting the formation of the inclusion complex was obtained by ^1^H-NMR, which has proved to be the most direct evidence in explaining the host–guest interaction of CDs and guest molecules [18,27]. The ^1^H-NMR of daidzein has a very low resolved spectrum in D_2_O due to its poor water solubility. We measured the ^1^H-NMR spectra of the CDs and the inclusion complexes of daidzein-β-CD, daidzein-Me-β-CD, and daidzein-HP-β-CD in D_2_O (see Supplementary data Figures S9–S14). The ^1^H-NMR spectra of the inclusion complexes showed all of the expected proton signals of daidzein and CDs, in agreement with significant solubilization. 

**Figure 6:**
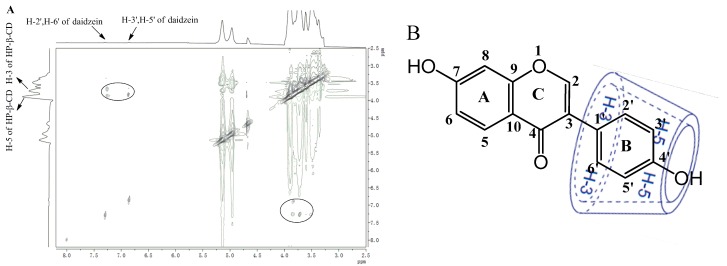
(**A**) ROESY spectrum of the daidzein-HP-β-CD inclusion complex in D_2_O at 25 °C; (**B**) Possible inclusion mode of the daidzein-HP-β-CD inclusion complex.

To understand the detailed inclusion fashion of daidzein-β-CD, daidzein-Me-β-CD, and daidzein-HP-β-CD, 2D ROESY NMR spectra were also measured. As shown in Figure 6A, the 2D ROESY NMR spectra of daidzein-HP-β-CD showed strong correlation signals between the inner H-3 and H-5 protons of the HP-β-CD and the daidzein protons. The spectra exhibited strong correlation signals between the H-3 protons in the HP-β-CD and the H-2′,6′ and H-3′,5′ protons in daidzein and between the H-5 protons in the HP-β-CD and the H-3′,5′ protons in daidzein, respectively. However, the spectra did not show any significant correlation signals between the H-3 proton in the HP-β-CD and the H-3′,5′ protons of daidzein. These data indicate that the HP-β-CD selectively includes the daidzein from the wide rim side to form the inclusion complex. It was also shown that daidzein should be encapsulated in the β-CD and Me-β-CD cavities in a similar way (see Supplementary data Figures S15 and S16). A study reported by Borghetti et al. [34] indicated that the formation of the inclusion complex between daidzein with CDs also occurred through the insertion of the B rings of daidzein into the CDs cavity, which is similar to our findings.

Based on these observations, together with the 1:1 stoichiometry, we deduced the possible inclusion modes of daidzein with CDs, as illustrated in Figure 6B.

### 2.6. Antioxidant Activity of Daidzein in Free and Complex Form

The evaluation of DPPH scavenging capacity was one of the most general methods to determine the antioxidant activities of different compounds [35,36]. DPPH had a strong absorbance at 517 nm due to the unpaired electron of nitrogen atom, which can accept an electron donated by the antioxidant compound. In this process, the DPPH was decolorized from purple to yellow which can be spectrophotometrically monitored from the changes to absorbance at 517 nm.

**Figure 7:**
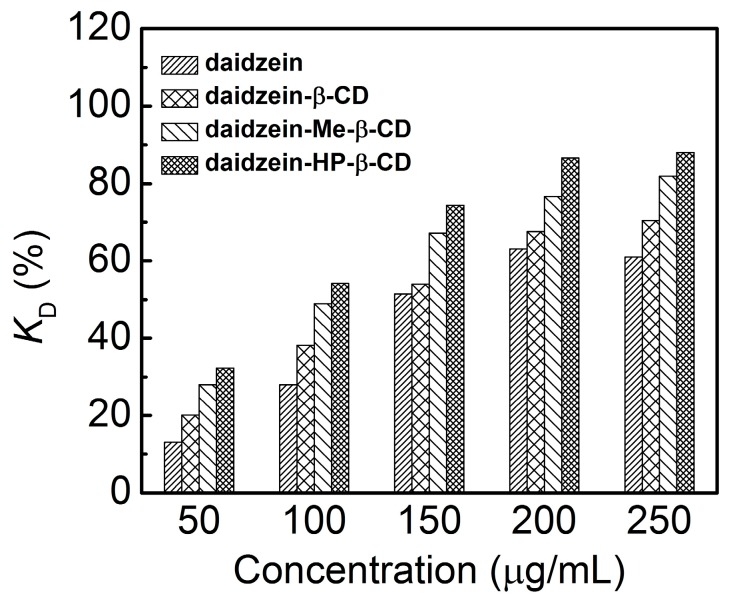
1,1-diphenyl-2-picryl-hydrazyl (DPPH) radical scavenging activities of the daidzein, daidzein-β-CD, daidzein-Me-β-CD, and daidzein-HP-β-CD inclusion complex.

Figure 7 showed a comparison of the DPPH radical-scavenging activity of daidzein, daidzein-β-CD, daidzein-Me-β-CD, and daidzein-HP-β-CD complexes. As shown in our findings, after complexation with CDs had occurred, the scavenging capability of daidzein increased significantly. The order was daidzein-HP-β-CD > daidzein-Me-β-CD > daidzein-β-CD, which indicates that the daidzein-CDs complexes have stronger DPPH radical-scavenging ability than the native daidzein have. The DPPH scavenging capacity of the antioxidant is closely related to its hydrogen-donating ability [37,38,39]. The increasing DPPH scavenging ability of daidzein could be attributed to the enhancement of its hydrogen-donating ability, caused by the complexation of CDs. When daidzein is complexed with CDs, one or more intermolecular hydrogen bonds form between daidzein and the CDs. This weakens the intramolecular hydrogen bonds of daidzein. Ultimately, the hydrogen-donating ability of daidzein is improved.

The stronger interaction between daidzein and HP-β-CD weakened the covalent bonds between hydrogen and oxygen in the hydroxyl groups, which in turn improved the hydrogen donation of the hydroxyl groups of daidzein. In contrast, the multiple methyl group substitutions of Me-β-CD impaired the hydrogen-bonding interaction between daidzein and Me-β-CD. This is unfavorable to the hydrogen-donating ability of daidzein. Ultimately, the DPPH scavenging ability of daidzein-HP-β-CD is stronger than that of daidzein-Me-β-CD, which is consistent with the binding ability of the three CDs. Therefore, we can conclude that the antioxidant property of daidzein-CDs is closely related their mode of binding.

## 3. Materials and Methods

### 3.1. Materials

Daidzein (>99%) was obtained from Aladdin Industrial Corporation (Beijing, China); β-cyclodextrin (β-CD, Mw = 1135 g/mol), (2-hydroxy)propyl-β-cyclodextrin (HP-β-CD, Mw = 1380 g/mol, average degree of substitution (DS) = 4.2), methyl-β-cyclodextrin (Me-β-CD, Mw = 1310 g/mol, average degree of substitution (DS) = 12.5) were purchased from Seebio Biotech, Inc. (Shanghai, China). 

### 3.2. Methods 

#### 3.2.1. Preparation of Daidzein/β-CD, Daidzein/Me-β-CD and Daidzein/HP-β-CD Inclusion Complexes 

Daidzein (0.6 mmol, 127 mg) was dissolved in 20 mL ethanol. CDs (β-CD, Me-β-CD, HP-β-CD, 0.3 mmol) were dissolved in 80 mL water, and then the CDs solutions were added to the daidzein solutions respectively. The mixture was sealed and stirred for 48 h. After evaporating the ethanol from the reaction mixture, the uncomplexed daidzein was filtered. The filtrate was frozen at −40 °C for 24 h and then lyophilized. The resultant powers were collected as the daidzein-CDs complexes. 

#### 3.2.2. Preparation of Daidzein/β-CD, Daidzein/Me-β-CD and Daidzein/HP-β-CD Physical Mixture

The physical mixture was prepared by mixing the powders in a 1:1 molar ratio of daidzein and CDs in an agate mortar. 

#### 3.2.3. Phase-Solubility Study

Phase-solubility studies were performed according to the method reported by Higuchi and Connors [26]. An excess amount of daidzein was added to 10 mL of aqueous solution containing different concentrations of β-CD, Me-β-CD, and HP-β-CD (from 0 mM to 5.0 mM). The mixtures were vigorously shaken with a shaking rate at 120 rpm in a water bath for 72 h at 25 °C. After reaching equilibrium, the samples were filtered through a 0.45 m hydrophilic membrane filter. All samples were prepared in triplicate. The concentration of daidzein in the filtrate was determined by a CARY-60 spectrophotometer (Varian, Palo Alto, CA, USA). The phase-solubility profiles were obtained by plotting the solubility of daidzein against the concentration of β-CD, Me-β-CD, or HP-β-CD. The apparent stability constants (Ks) were calculated from phase-solubility diagrams according to the following equation:

(1) $$K_{s}=\frac{\mathrm{Slope}}{S_{0}\left(1-\mathrm{Slope}\right)}$$

where $S_{0}$ is the solubility of daidzein at 25 °C in the absence of cyclodextrins and slope means the corresponding slope of the phase-solubility diagrams.

#### 3.2.4. Stoichiometry Determination: Job’s Method

The continuous variation method was performed in order to confirm the stoichiometry of the complex. The sum of the concentration of both components was kept constant ([daidzein] + [CDs] = 1 × 10^−4^ M) whilst the molar fraction of daidzein (R = [daidzein]/[daidzein] + [CDs]) was varied from 0.0 to 1.0. After stirring for 48 h, the UV-vis spectra were measured and the difference in the absorption between that in the presence (A) and absence of CDs (A_0_), ΔA = A − A_0_, was plotted against the molar fraction R. The host–guest ratio of the complex can be determined at the stoichiometric ratio. 

#### 3.2.5. Powder X-ray Diffraction (XRD)

Monochromatic Cu Ka radiation (wavelength = 1.54056 Å) was produced by a D/MAX 2500V/PC X-ray diffractometer (Rigaku Americas Corporation, Tokyo, Japan). The powders of samples were packed tightly in a rectangular aluminum cell. The samples were exposed to the X-ray beam. The scanning regions of the diffraction angle, 2θ, were 5–70°. Duplicate measurements were made at ambient temperature. Radiation was detected with a proportional detector.

#### 3.2.6. Thermal Analyses

Thermogravimetric (TG) and differential scanning calorimetry (DSC) measurements were performed with a DTG-60AH (Shimadzu, Kyoto, Japan) instrument, at a heating rate of 10 °C/min from 30 °C to 400 °C in a dynamic nitrogen atmosphere (flow rate = 70 mL/min).

#### 3.2.7. Scanning Electron Microscopy (SEM)

SEM photographs were determined on a TESCAN VEGA II. (Tescan Corportion, Brno, Czekh) The powders were previously fixed on a brass stub using double-sided adhesive tape and then were made electrically conductive by coating, in a vacuum with a thin layer of gold for 30 s and at 20 W.

#### 3.2.8. ^1^H-NMR and 2D NMR

The ^1^H-NMR and 2D ROESY was all recorded on a BRUKER AVANCE 600 NMR spectrometer (Bruker Corporation, Karlsruhe, Germany) at 25 °C. Deuterium oxide (D_2_O) was used as the solvent. Chemical shifts were referenced to the solvent values (4.70 ppm for HOD).

#### 3.2.9. DPPH Radical-Scavenging Capacity 

The antioxidant activity was measured by the scavenging of the stable free-radical DPPH, which showed a characteristic absorbance peak at 517 nm in ethanol. The addition of an antioxidant resulted in a decrease in the absorbance proportional to the concentration and antioxidant activity of the compound itself [40,41].

An ethanolic solution of the radical DPPH was prepared and protected from light. Daidzein-β-CD, daidzein-Me-β-CD or daidzein-HP-β-CD samples of different concentrations were added to DPPH ethanolic solution. DPPH free-radical scavenging by the daidzein-CDs inclusion complexes and native daidzein were investigated according to the method of Wang et al. [42]. Briefly, DPPH solutions (2.0 mL) in ethanol (2 × 10^−4^ mol/L) and 2.0 mL of tested samples with various concentrations were mixed in the tubes. Then, the mixture was incubated for 60 min in the dark at 30 ± 1 °C. The absorbance was measured at 517 nm in CARY-60 UV-vis spectrometer (Varian, Palo Alto, CA, USA). The lower absorbance of the reaction mixture indicated higher free radical scavenging activity. The DPPH scavenging effect (K_D_) was calculated using the following equation:

(2) $$K_{D}=\left(\frac{A_{0}-\left(A_{i}-A_{j}\right)}{A_{0}}\right)\times 100\%$$

where A_0_ A_i_ was the absorbance in the presence of the samples and A_j_ was the absorbance of the samples alone.

## 4. Conclusions

The inclusion complexes of daidzein with β-CD, Me-β-CD and HP-β-CD were prepared and characterized by phase-solubility, XRD, DSC, SEM, and antioxidant studies. The phase-solubility, XRD, DSC and SEM studies confirmed that daidzein can form inclusion complexes with three kinds of CDs, and the ratio between the host–guest molecules is 1:1. Furthermore, the solubility of daidzein was improved due to the formation of the inclusion complex. The 2D ROESY and ^1^H-NMR analyses show that the B ring of daidzein is the part of the molecule that is most likely inserted into the cavity of HP-β-CD, thus forming an inclusion complex. Antioxidant activity studies showed that the antioxidant performance of the inclusion complexes was better than that of the native daidzein, and the daidzein-HP-β-CD inclusion complex was the most effective form. Given the easy preparation and environmentally friendly process of creating daidzein-CDs inclusion complexes, it is a promising way to design a novel formulation of daidzein for herbal medicine or healthcare products.

## Supplementary Materials

Supplementary Materials are available online.
