# Supplementary material for: Studies on the Inclusion Complexes of Daidzein with β-Cyclodextrin and Derivatives
Source: Molecules. 2017 Dec 8;22(12):2183. doi: 10.3390/molecules22122183 (PMC6149782; doi:10.3390/molecules22122183)
Supplement: Supplementary file 1 [file molecules-22-02183-s001.pdf]

Supplementary Data

Studies on the inclusion complexes of daidzein with β-cyclodextrin and derivatives

Shujing Li[1*], Li Yuan[1], Yong Chen[2], Wei Zhou[1] and Xinrui Wang[1]

[1] Beijing Advanced Innovation Center for Food Nutrition and Human Health/Department of Chemistry, School of Science, Beijing Technology and Business University, Beijing 100048, PR China;

[2] Key Laboratory of Photochemical Conversion and Optoelectronic Materials, Technical Institute of Physics and Chemistry, Chinese Academy of Sciences, Beijing 100190, PR China

* Correspondence: lishujing@mail.ipc.ac.cn; Tel.: +86-10-6898-5573

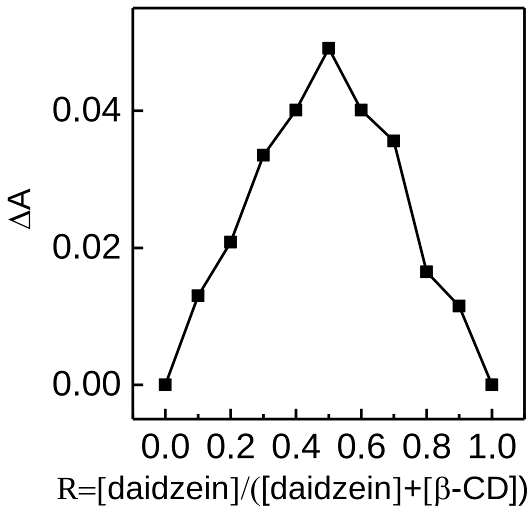

Figure S1 Job's continuous variation plot of the daidzein-β-CD complex

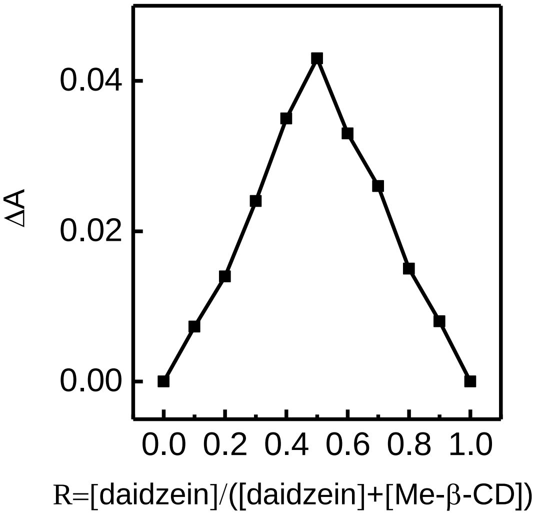

Figure S2 Job's continuous variation plot of the daidzein-Me-β-CD complex

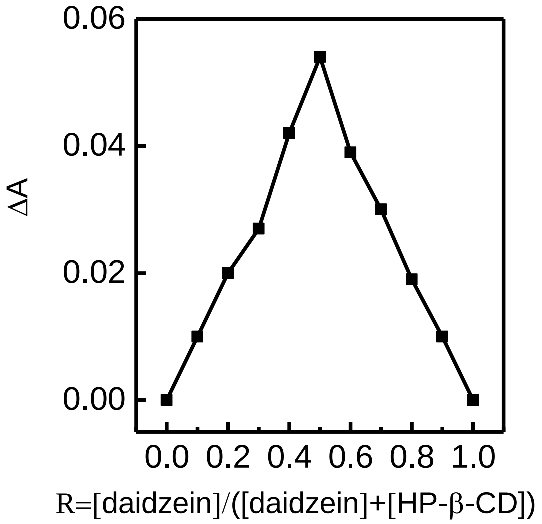

Figure S3 Job's continuous variation plot of the daidzein-HP-β-CD complex

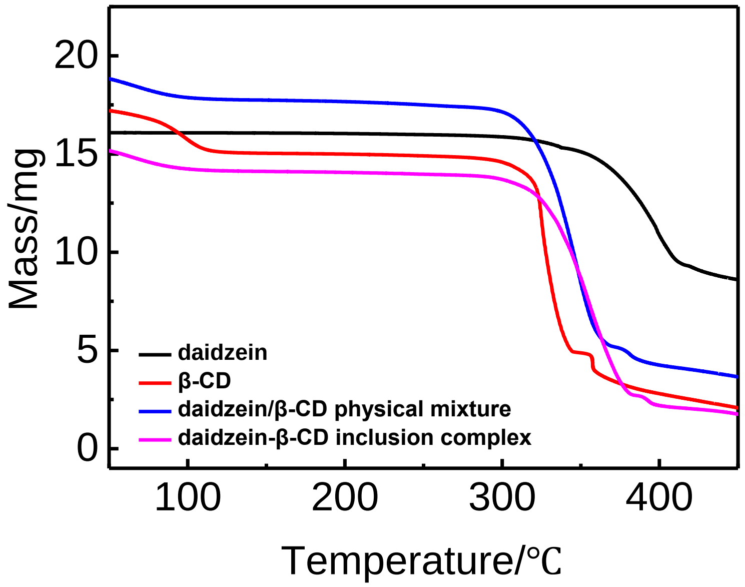

Figure S4 TG curve of daidzein, β-CD, daidzein/β-CD physical mixture, and daidzein-β-CD inclusion complex.

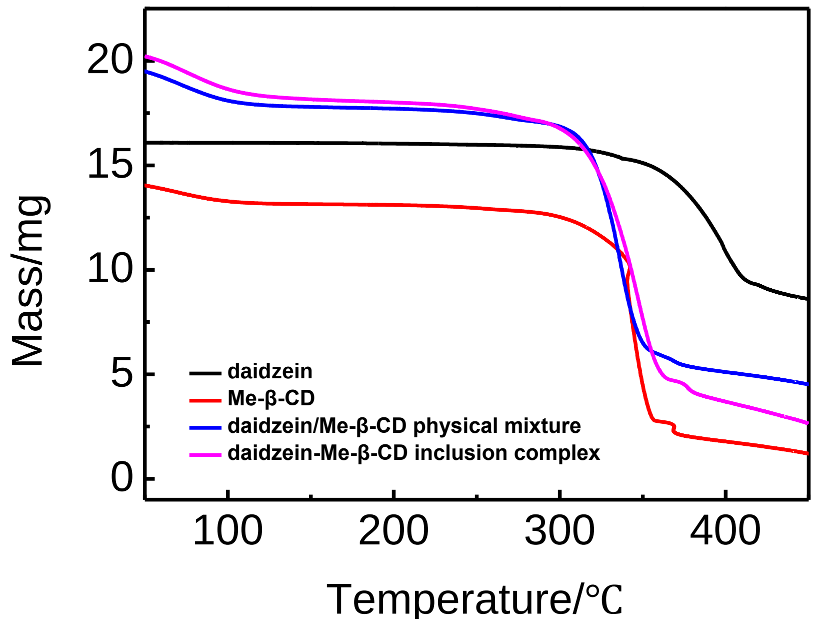

Figure S5 TG curve of daidzein, Me-β-CD, daidzein/Me-β-CD physical mixture, and daidzein-Me-β-CD inclusion complex.

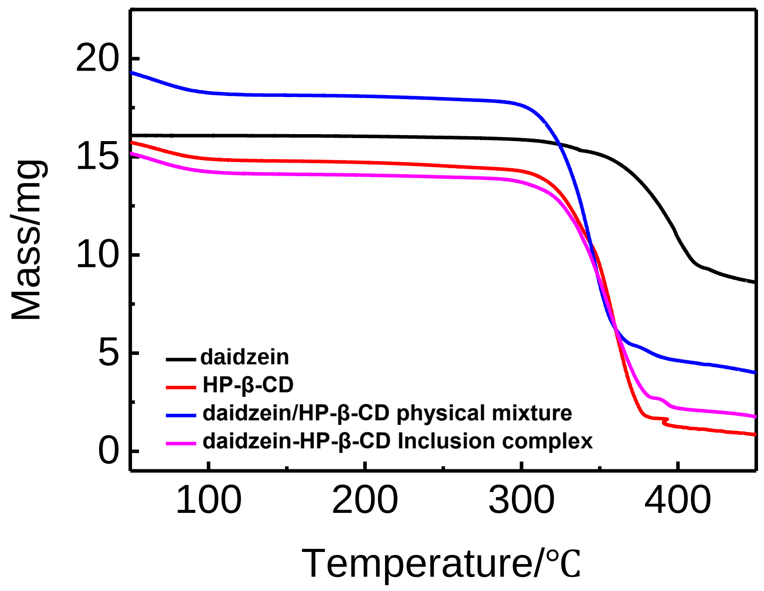

Figure S6 TG curve of daidzein, HP-β-CD, daidzein/HP-β-CD physical mixture, daidzein-HP-β-CD inclusion complex.

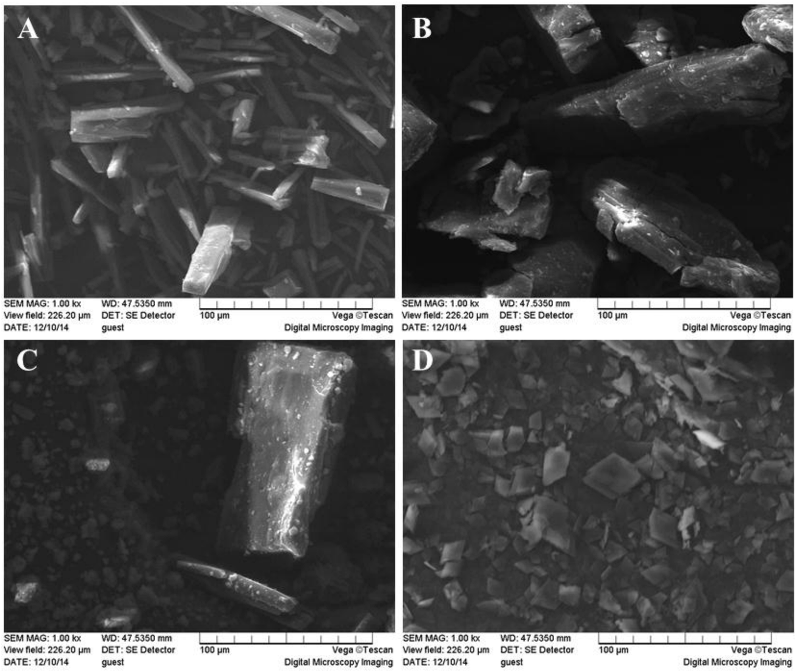

Figure S7 Scanning electron microphotographs: (A) daidzein, (B) β-CD, (C) daidzein/β-CD physical mixture, (D) daidzein-β-CD inclusion complex.

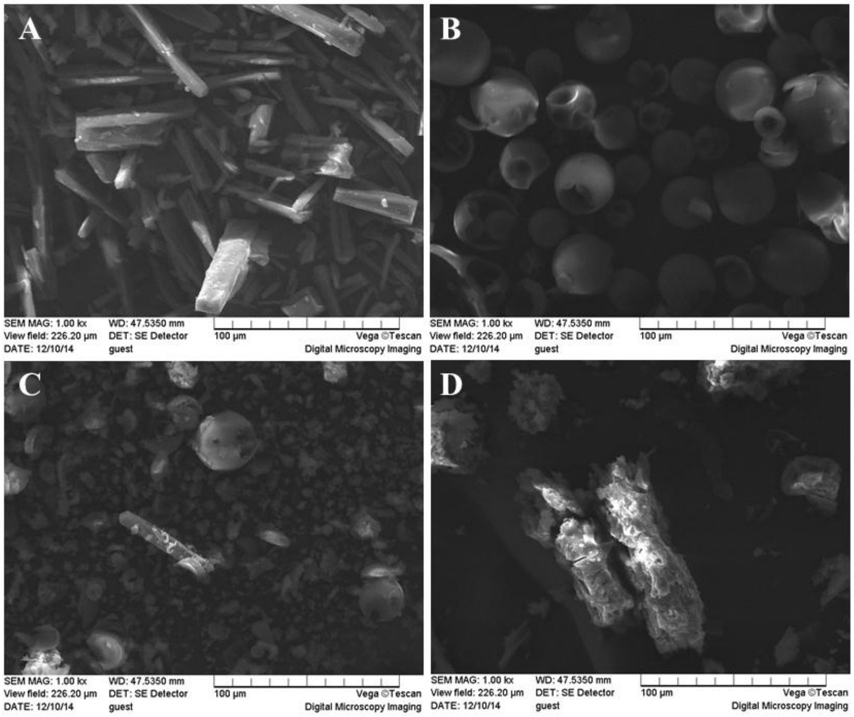

Figure S8 Scanning electron microphotographs: (A) daidzein, (B) Me-β-CD, (C) daidzein/Me-β-CD physical mixture, (D) daidzein-Me-β-CD inclusion complex.

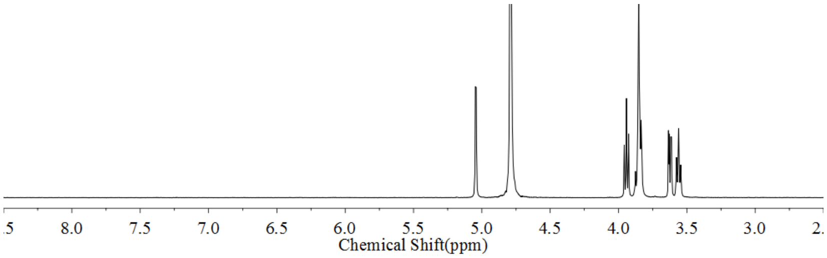

Figure S9 ^{1}H NMR spectra of β-CD in D_2O at 25 ◦C

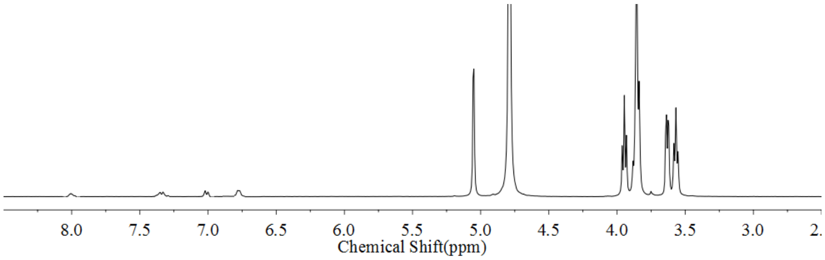

Figure S10 ^{1}H NMR spectra of daidzein-β-CD inclusion complex in D_2O at 25 ◦C

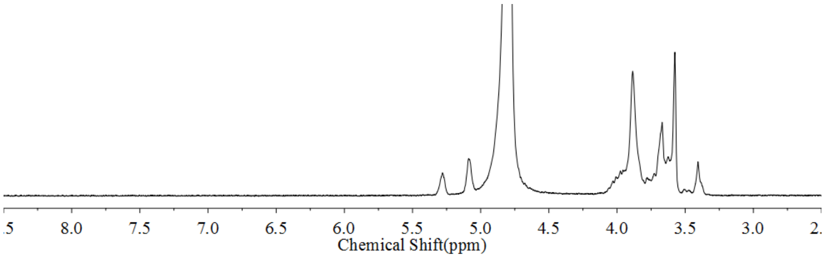

Figure S11 ^{1}H NMR spectra of Me-β-CD in D_2O at 25 ◦C

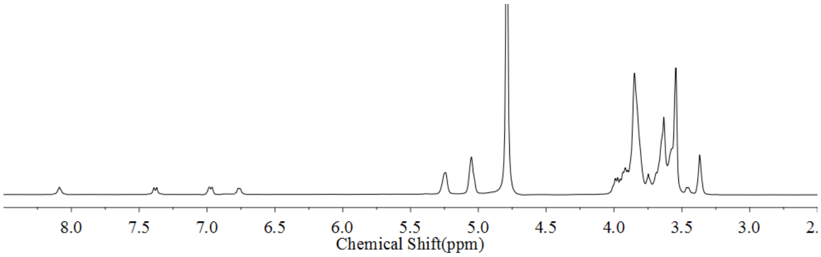

Figure S12 ^{1}H NMR spectra of daidzein-Me-β-CD inclusion complex in D_2O at 25 ◦C

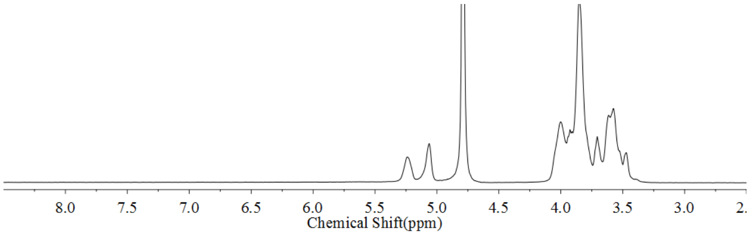

Figure S13 ^{1}H NMR spectra of HP-β-CD in D_2O at 25 ◦C

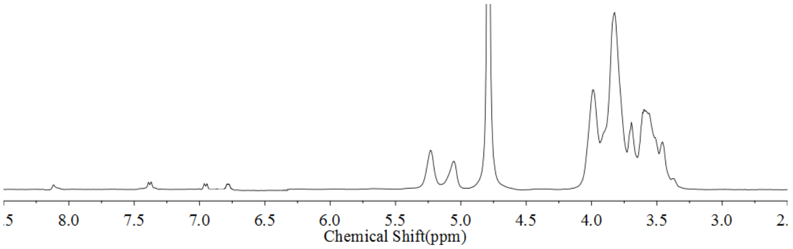

Figure S14 ^{1}H NMR spectra of daidzein-HP-β-CD inclusion complex in D_2O at 25 ◦C

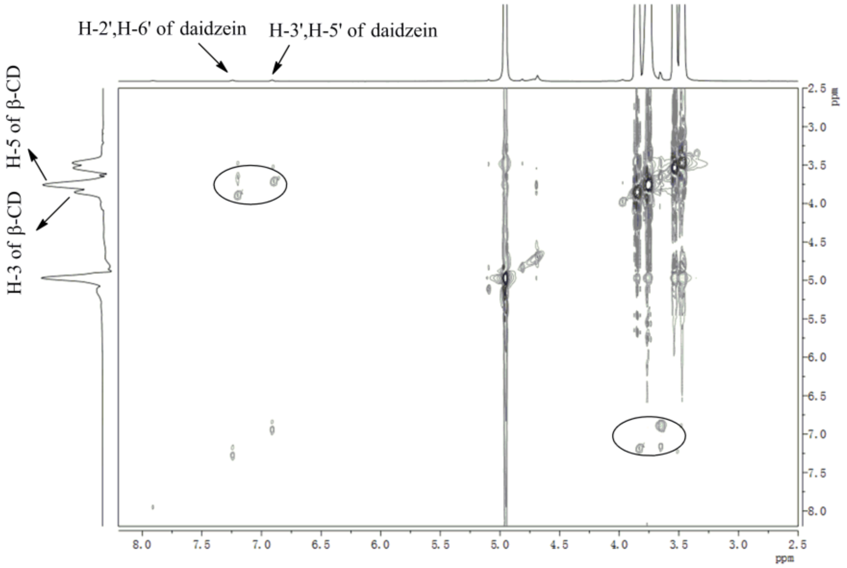

Figure S15 ROESY spectrum of daidzein-β-CD inclusion complex in D_2O at 25 ◦C

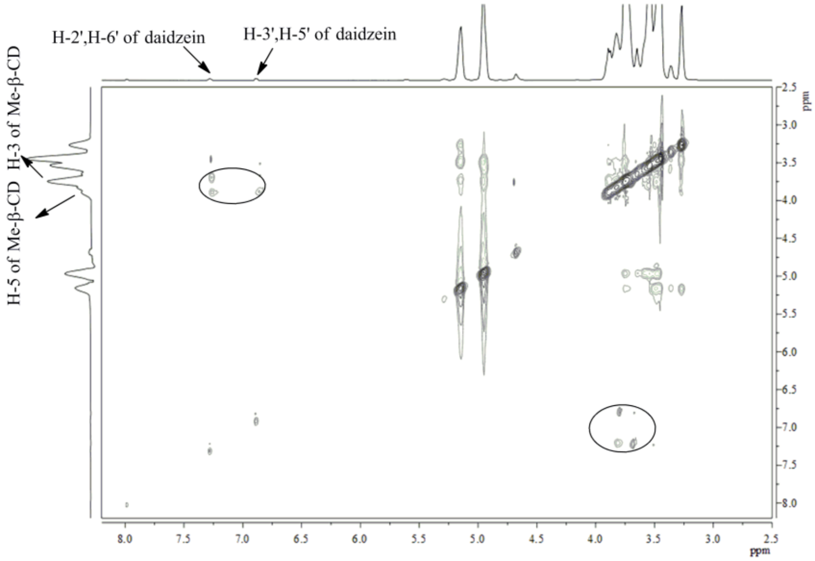

Figure S16 ROESY spectrum of daidzein-Me-β-CD inclusion complex in D_2O at 25 ◦C
